# Congenital Splay Leg Syndrome in Piglets—Current Knowledge and a New Approach to Etiology

**DOI:** 10.3389/fvets.2021.609883

**Published:** 2021-02-26

**Authors:** Toni Schumacher, Monika Röntgen, Steffen Maak

**Affiliations:** Institute of Muscle Biology and Growth, Leibniz Institute for Farm Animal Biology, Dummerstorf, Germany

**Keywords:** congenital splay leg syndrome, piglet, myofibrillar hypoplasia, HOMER1, FBXO32

## Abstract

The porcine congenital splay leg syndrome (PCS), even though being of transient nature, is still one of the most important causes for piglet losses due to its high incidence and mortality. Although, described decades ago, the pathogenetic mechanism is still elusive. Numerous, mostly descriptive studies characterized the syndrome at clinical, histological and cellular levels but resulted in a highly diverse picture of the syndrome. Broad variability in phenotypical expression and, in case of proper care, the rapid recovery of affected animals complicated a systematical analysis of the underlying pathogenesis. Although, several environmental factors were discussed as potential causes of PCS, most of the evidence points to a hereditary basis of PCS. Nevertheless, only few of the suggested candidate genes from transcriptome and mapping analyses, like F-box protein 32 (FBXO32), could be confirmed so far. Only recently, a genome wide association study revealed genomic regions on five porcine chromosomes and named a number of potential candidate genes, among them homer scaffold protein 1 (HOMER1). This new candidate—a cellular scaffold protein—plays a role in a plethora of cellular signaling cascades, and is not only involved in skeletal muscle differentiation but also critical for muscular function. In this review, we critically elucidate the current state of knowledge in the field and evaluate current achievements in the identification of the pathogenetic mechanism for the syndrome.

## Introduction

The porcine congenital splay leg syndrome (PCS) or spraddle leg syndrome is generally considered as one of the most frequent developmental defects in piglets (1). This condition is characterized by a lateral abduction of the hind limbs in newly born animals while the forelegs are affected only in severe cases. This leads to an impaired ability of the piglets to walk or even stand and increases the risk of being crushed by the sow or to die of starvation. Losses among affected piglets are high and discussion is still ongoing whether muscle function remains impaired throughout life of surviving piglets (2, 3). Consequently, PCS is highly relevant for the pig industry due to economic reasons as well as for animal welfare issues. The splay leg syndrome is generally transient and animals will recover within a few days if treated properly (4). Tying the affected hind legs immediately after birth increases mobility, ensures proper nutrition and is mostly sufficient to decrease mortality rates significantly (5).

Despite more than 50 years of research in different countries with large scale and intensive pig production like the USA (3), Canada (6), Germany (7), Australia (8), Czech Republic (9, 10), neither the etiology nor the pathogenic mechanisms of the splay leg syndrome are fully understood. Histological, histochemical and biochemical analyses were mainly performed from the 1960's to the 1990's describing more or less typical features of delayed muscle maturation (11, 12). Early on, herd level analyses suggested a hereditary predisposition due to breed specific incidence rates and effects of mated boars (13). With increasing availability of methods for genome-wide, molecular analyses, studies aimed at the elucidation of genetic causes of PCS at both, transcriptome and genome level since the early 2000's (14–16). However, the transient nature as well as the phenotypic variability of PCS, and the snap-shot-like picture provided by transcriptome analyses led to limited insight into the pathological mechanisms although, a number of functional candidates were provided for further analyses. Only recently, a genome wide association study (GWAS) revealed different genomic regions on five porcine chromosomes of which some contain promising candidate genes for PCS (17).

This review provides a comprehensive update on the current knowledge about PCS and highlights new approaches for future research.

## Incidence and Mortality

Reports on the incidence of PCS differ considerably depending on breed, scale of farms, and country. Early data from large-scale production units in East Germany indicated a proportion of 22–33% affected piglets among all piglets born alive. Almost 5% had to be culled (18, 19). In contrast, incidences of 5–7.5% were reported under similar production regimens by other authors while between 21 and 36% of all litters were affected (20, 21). In a more recent investigation 0.2% splay leg piglets were counted among all piglets born alive whereas 1.2% of all piglets were affected in German Landrace and their crosses with Pietrain (22). Between 0.12 and 1.42% of all born piglets were affected by the syndrome in Large White and Landrace in Great Britain in 1972. Similar results were stated for other European countries, Canada and Australia (23). Nowadays, it is assumed that the general incidence ranges from 1 to 8% worldwide (24), although newer, more comprehensive records are not available. The results also clearly indicate that large pig facilities are more often affected. While housing and breeding conditions certainly play a significant role in splay leg incidence, there is also clear evidence for hereditary factors causing PCS.

Several studies found differing incidences clearly related to genetic background and sex. Already in 1971 it was shown that male piglets are affected disproportionately high and that the gender ratio is roughly 2:1 favoring incidence in male vs. female (13) which was not consistently confirmed by others (21, 25, 26). Effects of litter size were also discussed but were not found significant in a larger study over several years (26). Beside the gender effect, there must be other hereditary factors since later it was also found that certain breeds tend to have higher incidences. For example, Landrace crossbred pigs had significantly higher proportions of splay leg piglets in comparison to progeny of Yorkshire and Duroc (26) and thus confirmed an earlier study (20). Landrace for example showed a nearly 10-fold higher incidence of PCS compared to Large White and hybrids were characterized by intermediate incidences (23). It is therefore reasonable to view congenital splay leg as a hereditary disease. With 45% of all recorded inherited defects, it even represents the largest group of hereditary birth defects in pigs (23, 27).

Due to the high incidence, splay leg syndrome was the second most common cause of piglet mortality (22%) with a general lethality of up to 50% according to recent studies (5, 28). The significance of this disease with regard to the economic losses and its negative effect on animal welfare and health is considerable and should not be underestimated (6, 13, 17).

## Phenotypic Characterization

### Clinical Signs and Treatment

**Figure 1:**
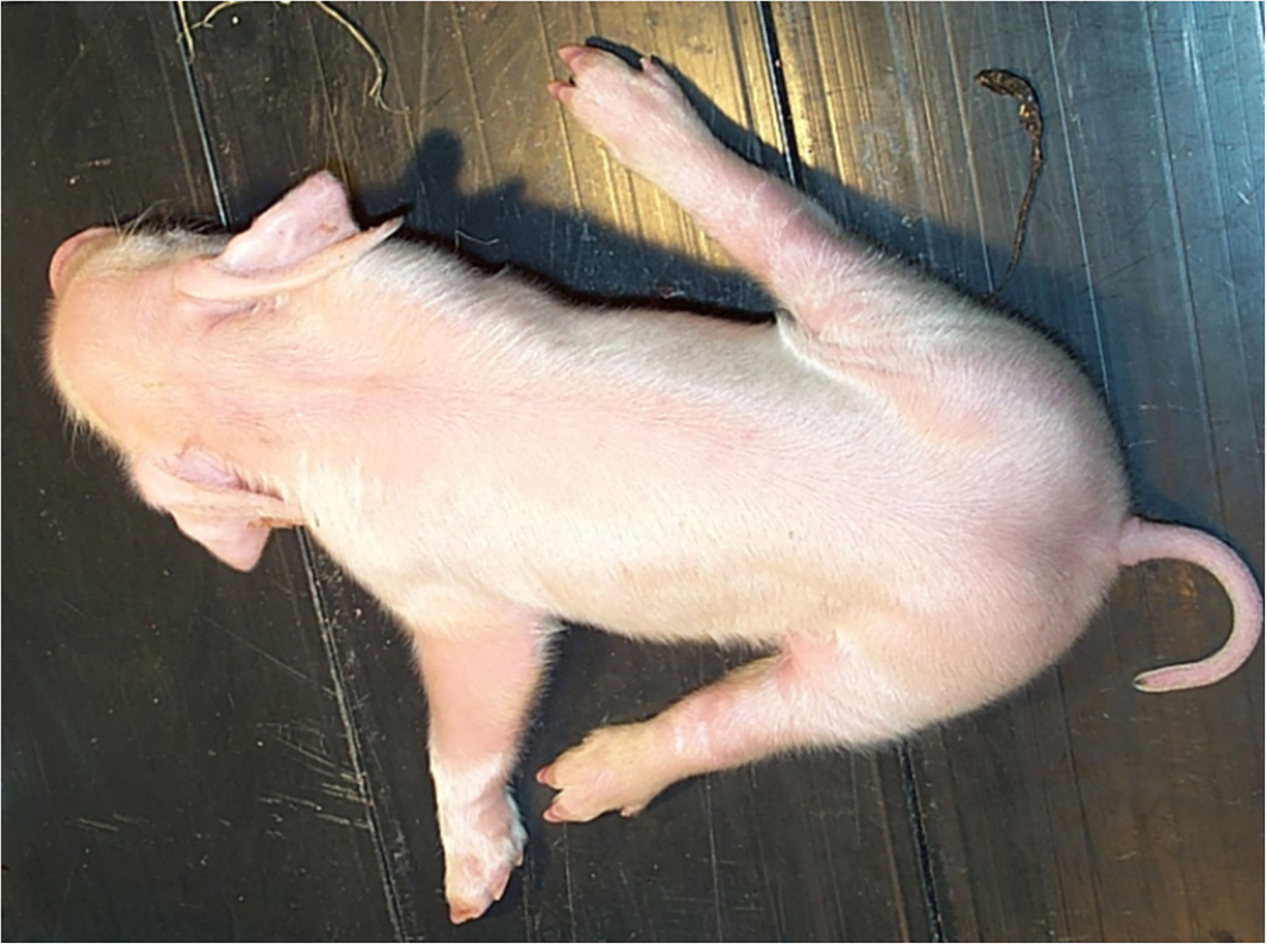
Typical phenotype of a splay leg piglet. The hind legs are splayed sideward impairing the ability to stand and walk. The degree of splaying differs and fore legs are affected in severe cases, too.

The clinical signs of PCS were first described in the late 1960's (29) and are visible directly after birth or within the 1st h of live. Affected animals exhibit a characteristic position of the hind limbs, which are deflected laterally and in severe cases, also the forelimbs are spread sideways (Figure 1). These signs differ in their expression and lead to difficulties or complete inability to stand and walk, which limits or even prevents suckling. In addition, affected piglets are at a high risk to be crushed by the sow. In cases with milder clinical signs, diseased piglets are able to overcome the critical period of postnatal paresis and apparent self-healing occurs within the 1st week of life (30). Permanent damage is not detectable and the splay leg piglets do not significantly differ in growth and phenotype from their healthy littermates (4). Common treatment methods of less affected animals are tying the hind limbs during the 1st days to improve overall mobility and to ensure proper nutrition by accessing the teats of the sow. With these simple but labor-intensive measures the chances for survival of the affected piglets increase significantly, even though a medical treatment is not available.

### Muscular Phenotype

Besides the obvious clinical signs, splay leg affected piglets show subtle histological findings. A histological myofibrillar deficiency in splay leg piglets leading to a muscular weakness was described alongside the syndrome's first scientific mention (29). Back then, Thurley et al. (29) introduced the term myofibrillar hypoplasia (MFH) to describe a histological pattern where only few numbers of small myofibers are found in the muscle section, which are also concentrated in a small part of the area enclosed by the endomysium. Due to rigorous investigation of a large number of different skeletal muscles, they also found MFH occurrence decreasing in the order hind limb > lumbar region > forelimb (29). Since recognized methods for thorough, objective histological studies were missing when PCS was first described, early studies on the extent of MFH might have been somehow biased by their subjective nature (2).

A more conclusive study, involving a total of 24 affected piglets and controls and an extensive set of histological methods, was capable of setting up a more comprehensive picture of PCS histology (10). Beside the known MFH, staining for glycogen, ATPase and acetylcholine esterase (ACHE) revealed the presence of impaired muscular differentiation in PCS-affected piglets. In particular, using acid pre-incubated ATPase-reaction, the authors found only small numbers of solitary type I fibers in PCS-affected samples. This points to a striking delay in conversion of type II to type I myofibers, a main component of postnatal muscle maturation. In addition, ACHE-staining showed clear differences between larger, well-organized motor endplates in controls compared to smaller and more chaotically organized ones in splay leg samples (10).

Due to high variation in fiber morphology, early studies were not able to quantify the amount of MFH reliably by measuring and comparing the mean muscle fiber areas. Furthermore, healthy neonatal piglets may have myofibers with a very small diameter that indeed resemble MFH, thus making it difficult to diagnose PCS based solely on histopathology in some cases (31).

Other studies defined the degree of MFH as distribution of muscle fibers per area unit instead measuring fiber diameters only. Modeling distributions from both, healthy and affected animals, showed clear differences in muscle fiber density, and size distribution (32). Development of semi-automatic measurement methods allowed evaluation of larger numbers of muscle fibers and led to establishment of MFH as a key feature in PCS-piglets. It is now accepted that a higher number of smaller, less matured myofibrils characterizes PCS. These changes are expressed to a different extent in different muscles (5, 16).

Earlier results also supported the notion that an incomplete maturity of the skeletal muscles in affected animals is present at the time of birth (12). The reason why hind leg muscles are predominantly affected might be the slightly earlier development of fore limbs during embryogenesis (33). This is in concordance with early results of Deutsch and Done (34) showing that the muscular phenotype of splay leg piglets corresponded to those of 80-day-old porcine fetuses (34).

There is also the agreement that piglets, which recovered from the disease, show no differences in histology compared to clinically healthy animals (7, 35).

### Cellular Phenotype

Already four decades ago, researchers employed electron microscopy to analyze skeletal muscle of PCS piglets at the ultrastructural level. Although, limited in numbers of investigated animals, striking differences between PCS-affected and control animals regarding the number, distribution and shape of cellular organelles were observed (7, 9). Again, the high variability of PCS-affected piglets prevented valid, general conclusions with regard to pathogenic mechanisms.

The most direct link between ultrastructural abnormalities and the pathology of PCS was the finding of disturbed morphology of the contractile elements. Morphologic disarray occurs in form of Z-discs with a streaming pattern in one or several adjacent myofibrils. Whole filaments seemed to be disarranged because myosin and actin were apparently misaligned. The affected sarcomeres appeared thin and often disrupted (9). Muscle fibers with extensive MFH contained differentiating myofibrils with incomplete or defective assembly of myofilaments (7). It is very comprehensible that such ultrastructural disarray leads to an impaired function of affected muscles.

Further studies showed that the sarcoplasmic reticulum was dilated and t-tubules were small and misshaped. Lysosomes and lipid droplets were found more frequently within the sarcoplasm while mitochondria were evenly distributed instead of being adjacent to the sarcolemma in normal piglets (36).

Atypical mitochondria were also observed in rabbits suffering from a syndrome with certain similarity to PCS. Mitochondria of affected animals were unevenly distributed and irregularly shaped. Sometimes mitochondria extended along the length of several sarcomeres (37).

Disarranged, misshaped mitochondria with probably impaired functional properties might therefore be responsible for the repeatedly observed phenomenon of splay leg muscles showing an increased accumulation of glycogen compared with the muscles of normal piglets. Here, the fibers that are lacking myofibrillar structure are filled with granular accumulations which were independently identified as glycogen stores (10, 34, 36, 38). Normally, fast depletion of glycogen takes place during the first 24 h after birth and muscle glycogen even decreased by 50% within the first 12 h after birth (39, 40). Thus, other mechanisms such as loss of contractile function due to structural disarray and the resulting decreased energy expenditure for physical activity, suckling and thermoregulation and/or impaired mitochondrial function might contribute to a reduced glycogen metabolism and development of higher numbers of glycogen granules.

Another intriguing hypothesis was an impairment of muscle innervation due to incomplete electrical isolation of primary motor neurons. Unfortunately, only a single study focusing on the nerval aspect of motor functions was published so far (41). According to this study, PCS-affected piglets suffer from loss of proper muscle innervation due to impaired Schwann-cell maturation leading to inefficient myelin sheathing. Extrapyramidal tracts and parts of the corticospinal tract were found largely unmyelinated in an analysis of the lumbar spinal cord of PCS-affected animals. The obturator nerve also showed a significant loss of myelin sheath thickness and a decreased overall axonal diameter while the number of axons was not changed (41).

### Biochemical Characteristics

Since histological features of PCS resemble that of degenerative myopathies, activities of muscle specific enzymes [alanine-aminotransferase (ALAT), aspartate-aminotransferase (ASAT), creatinine-phosphate-kinase (CPK), and lactate dehydrogenase (LDH)] in blood plasma were measured as markers of a modified muscle cell membrane permeability. An early study suggested increases in ASAT, ALAT CPK, and LDH plasma levels as characteristic for PCS (42) but further studies on plasma and supernatants from tissue homogenates were inconclusive (43, 44). Measurements of other enzymes (e.g., Ca^2+^-ATPase in nervous system, catalase as key enzyme of peroxide depletion) as well as of metabolites and serum proteins (glucose, α2-macroglobulins, and ascorbic acid) sometimes revealed significant differences between PCS and control groups. However, the results could not be convincingly related to a pathogenic mechanism for the syndrome (45–47). Changes in K^+^-distribution are also in line with a supposed loss of cellular membrane integrity (42). However, the collective data suggested, that PCS was different from all myopathies described in other species.

Splay leg samples showed reduced DNA amounts and reduced protein: DNA ratios in certain muscles of the hind leg (11, 12). This was interpreted as delayed skeletal muscle development in low-birthweight PCS piglets. Inefficient or absent muscle innervation could be a reason for the delayed maturation or degeneration leading to muscular dysfunction. An increased Ca2+- and Na+-content measured in certain skeletal muscles of the hind limbs of splay leg piglets, supported the assumption of a neuromuscular background of the disease (11). In addition, the nutritional aspect was analyzed in detail with regard to mineral nutrients and trace elements, though significant differences in PCS compared to healthy piglets were found in neither of the tested tissues (43).

Based on an early finding that dietary supplementation of choline to pregnant sows seemed to prevent the occurrence of splay leg animals (6), later investigations focused on choline metabolism. Insufficient choline supply was considered as either a cause for a lack of choline phospholipids as membrane components or to be causing disorders in signal transduction by a lack of acetylcholine as transmitter. Even a modified degradation of acetylcholine was considered (44). This was supported by positive effects of the administration of the acetylcholine esterase blocker neostigmine-methylsulfate on the incidence of PCS (21). However, these results have not been confirmed in other studies (44, 48, 49). In contrast, it was shown that PCS-affected animals showed significantly increased acetylcholine esterase activities in cerebellum, brain stem and spinal cord (50). Moreover, activity of choline acetyl transferase, which is responsible for synthesis of acetylcholine, was instead reduced in nerve tissues of splay leg affected animals (51). It seems that both, synthesis and degradation of acetylcholine, are disturbed in splay leg piglets and a probable disarray in muscle innervation might be at least partially involved in the expression of clinical signs.

Taken together, biochemical analyses of tissues and plasma from PCS piglets underline the heterogeneity of the syndrome as it was already noted for muscular and cellular phenotypes. Still though, data consistency suffers from low sample numbers, non-standardized phenotypes, and different time points of sampling.

## Genetic Characterization of the Splay Leg Syndrome

Even a decade after the initial description (29), PCS was often considered a consequence of insufficient intrauterine nutrition (6, 20). Nevertheless, numerous investigations presented convincing evidence for a genetic background of PCS (26, 52–54). Because environmental factors exerted a major influence on incidence and severity of the syndrome (55), it was noted that an environmental “threshold” seemed to be necessary for clinical manifestation of PCS (18). Quantitative, genetic analyses revealed an oligo- or polygenic heredity with an estimated heritability of up to 0.47 (22, 54).

Initial molecular genetic approaches isolated expressed sequence tags (ESTs) unique to splay leg piglets in order to identify potential candidate genes (14, 56). Subsequent candidate analyses however, could not establish a systematic link to the syndrome (57, 58). More targeted analyses became feasible with increasing knowledge of the porcine genome and transcriptome. Since it was not clear, whether the observed MFH was a result of delayed muscle growth and differentiation or an atrophic process, structural proteins and atrophy markers were investigated (5). The authors found several muscular structural proteins like the MYH genes MYH1, MYH4, and MYH7 to be down-regulated in PCS transcriptomes. The atrophy marker FBXO32 (MAFbx) was significantly overexpressed along with a clearly decreased expression of the yet unknown P311 protein (5), later identified as trans-differentiation regulator NREP which is known to be responsible for cell differentiation (59). The question whether muscle atrophy was cause or effect of impaired muscle maturation remained unanswered. Moreover, the differential expression of FBXO32 and P311 was not confirmed in an independent study (60).

When technological advances allowed the analysis of whole transcriptomes, the idea was quite tempting to deduce affected signaling networks just from the identified differentially expressed genes (DEG). The first microarray-based analysis on three hind limb muscles (M. sartorius, M gracilis, and Mm. adductores) of newborn PCS piglets and controls revealed only about two dozen DEGs with just a handful of genes with differential expression in all investigated muscles (15). These commonly affected genes were for example part of a universal expression response to multiple stressors (DDIT4), involved in regulation of protein degradation (SQSTM1), acting as transcriptional co-activator (SSRP1), transcription factor (MAF), or are involved in differentiation processes (ENAH). All of them appear quite coherent regarding the given clinical and histological phenotype. DDIT4 for example, known to act as an mTOR inhibitor, has been identified as a key regulator of muscular mass. Since muscle mass is in result dependent on the net balance of constant protein biosynthesis and protein degradation, impaired mTOR activity can shift the balance toward decreased protein synthesis and increased ubiquitin-dependent proteasome activity and autophagy (61). Increased expression of SQSTM1 also fits the frame due to its role as an autophagy adaptor protein being involved in shuttling ubiquitinated proteins toward the proteasome (62). Besides the aspect of muscle atrophy, also the impaired muscle differentiation observed in PCS-affected animals was backed up by certain DEGs. SSRP1 for example is one compound of the FACT (Facilitates chromatin transcription) complex that is responsible for expression of critical genes in muscle differentiation which it facilitates in a myogenin-dependent manner. It is needed during the early steps of muscle cell differentiation but it can only interact with its target genes in the presence of myogenin (63). Also, MAF, a well-known transcription factor involved in cellular differentiation, plays, in feedback with other transcription factors of the MRF (myogenic regulatory factor) family, an important role in myogenic differentiation (64). Its differential expression found in PCS-affected vs. control animals therefore supports the histological picture of delayed muscle differentiation. Another example of DEGs initially found using micro-array approaches was ENAH. This protein is involved in nucleation and polymerization of actin and is therefore involved in virtually every aspect of cellular functions which require actin reorganization [i.e., cell migration, cell-cell or cell-matrix adhesion, and cell differentiation (65)].

RNA-sequencing of unspecified hind leg muscle samples of 2-day old PCS and control piglets later revealed more than 1,000 genes with unique expression in one of both groups and 555 DEGs (16). The large number of DEGs allowed pathway enrichment analyses and identified a number of cellular signaling pathways including steroid biosynthesis, PI3K-Akt signaling, the phagosome, and ECM-receptor interaction. In the end, all of the significantly enriched pathways play crucial roles in differentiation and cellular growth (steroid biosynthesis), tissue remodeling and inflammation (phagosome), tissue and organ morphogenesis as well as maintenance of cell and tissue structures (ECM-receptor interactions), while the PI3K-Akt-signaling pathway regulates the most fundamental cellular functions like growth, transcription and survival. It was concluded that PI3K-Akt signaling is somehow suppressed in PCS leading to dephosphorylation of FOXO transcription factors toward overexpression of FBXO32 (16) which has been linked to muscle atrophy in PCS before (5). Although, the results of transcriptome analyses provided starting points for subsequent candidate investigations, they comprise only a snapshot of the gene activity in a specific tissue at the very moment of sampling and do not allow for discriminating between cause and consequence of the syndrome (Table 1). It should be kept in mind that few key nodes control many cellular pathways where minute changes in activity could affect a plethora of subsequent regulation cascades, while their own expression levels might change only marginally and oftentimes undetectably. Thus, such key players might be overlooked in these experimental approaches.

**Table 1:** Selected candidate genes for porcine congenital splay leg identified by gene expression analyses.

**Candidate gene (official symbol)**	**Gene name**	**Biological function/pathway**	**Analyzed tissues**	**Results**	**References**
MAF	MAF bZIP transcription factor	Transcription factor, DNA binding	Mm. adductores, gracilis, sartorius	Down in PCS	(15)
MyoD (MYOD1)	Myogenic differentiation 1	Myoblast differentiation	Mm. semitendinosus, longissimus dorsi, gastrocnemius, biceps femoris	Down in PCS at protein level	(66)
MyoG	Myogenin				
P311 (NREP)	Neuronal regeneration related protein	Myogenic differentiation	Mm. semitendinosus, longissimus dorsi, gastrocnemius;	Down in PCS	(5)
MAFbx (FBXO32)	F-box protein 32	Atrophy, autophagy, ECM receptor interaction		Up in PCS	
			Mm. adductores, gracilis, sartorius	Not different between PCS and control	(60)
			Mm. semitendinosus, longissimus dorsi, gastrocnemius, biceps femoris	Up in PCS at mRNA and protein levels	(66)
			Hind leg muscles	Up in PCS	(16)^*^
ITGA2B	Integrin subunit alpha 2b	Myogenic differentiation		Up in PCS	
ITGAV	Integrin subunit alpha V			Down in PCS	
GHR	Growth hormone receptor	Growth hormone signaling		Up in PCS	
SQSTM1	Sequestosome 1	Autophagy, ubiquitin / proteasome system	Mm. adductores, gracilis, sartorius	Up in PCS	(15)
SSRP1	Structure specific recognition protein 1	Myogenic differentiation		Up in PCS	
DDIT4	DNA damage inducible transcript 4	Autophagy, apoptosis mTOR pathway		Up in PCS	
ITGA5	Integrin subunit alpha 5	Myogenic differentiation	Mm. adductores, gracilis, sartorius	Up in PCS	(67)
ZDHHC9	Zinc finger DHHC-type palmitoyltransferase 9	Ras signaling	Mm. adductores, gracilis, sartorius	Up in PCS	(68)

* *a total of 555 genes differentially expressed between PCS and control piglets have been identified*.

Consequently, mapping of genetic variation linked to the PCS was applied in parallel experiments. First, a quantitative trait locus (QTL) analysis in a resource population based on microsatellites identified two suggestive QTL-intervals on SSC5, and SSC11 linked to the PCS phenotype. In these large QTL-intervals, 11 potential candidate genes were identified (69). Since NFYB (Nuclear transcription factor Y, beta), was found to be differentially expressed before (14) and is located within the QTL on SSC5 it underwent scrutiny with regard to its genomic sequence and expression. Even though a sex difference in NFYB expression was observed, a relationship to PCS was finally excluded (58). No further candidate genes from this first QTL screen were considered later on. Although, QTL mapping promised identification of potentially causal variation at genome level, it was hindered by the need for sufficiently large resource populations and the low density of available markers comprising microsatellites in the beginning. This explains that no further studies have been published.

Only recently, a genome wide association study was performed in a case/control design in five different pig populations that gained new insights (17). Following this approach, seven chromosome-wide significant SNPs were identified on five porcine autosomes (SSC1, SSC2, SSC7, SSC15, and SSC16) but none of the SNPs reached genome-wide significance level. This might be attributed to the limited population size as well as to the rather strict correction for multiple testing. Notably, SSC5 and SSC11 identified by Schwarz (69) as QTL-harboring autosomes before, were not confirmed by this study. The seven SNPs were located in a distance between 0 and roughly 200 kb to a neighboring gene. Of these seven, two SNPs were located only 18 kb from or directly within homer scaffold protein 1 (HOMER1) on SSC2, and two further SNPs in an intergenic region on SSC15, 97 and 120 kb apart from transition protein 1 [TNP1; (17)]. Although, the latter two SNPs seem not to relate to an obvious candidate gene for PCS it is conceivable that a not yet annotated non-coding gene might be the carrier of the respective SNPs. More interestingly, two SNPs are located close to and within HOMER1, a gene previously linked to muscle related processes. Even though the specific function of HOMER1 has not been studied in pigs sufficiently, it is already known to be involved in muscle differentiation and calcium homeostasis (70), leading to myopathy in mice when expression is lost (71) and exhibited lower expression in a mouse model of Duchenne's muscular dystrophy (72). A follow up study (73) focused directly on the sequence of porcine HOMER1, defined the promoter regions and analyzed a total of 21 SNPs in the same population used in their previous analysis (17). Twelve of the SNPs were significantly associated with PCS making HOMER1 a promising candidate for PCS (Table 2).

**Table 2:** Structural analyses of candidate genes for PCS.

**Candidate gene**	**Gene name**	**Biological function/pathway**	**Results**	**References**
CDKN3	Cyclin dependent kinase inhibitor 3	Cell cycle, carcinogenesis	No association of 12 single nucleotide polymorphisms (SNPs) with PCS	(57)
ITGA5	Integrin subunit alpha 5	Myogenic differentiation	No association of a SNPin the 3‘-UTR^*^ with PCS	(67)
ZDHHC9	Zinc finger DHHC-type palmitoyltransferase 9	Ras signaling	No variation between PCS and control	(68)
HOMER 1	Homer scaffold protein 1	Muscle development, glycogen metabolism	12 of 19 SNPs related to PCS, 1 SNP changes activity of an internal promoter	(73)

* *3‘-UTR: 3‘untranslated region*.

In a completely different approach, a large-scale ENU (N-ethyl-N-nitrosourea) mutagenesis screen was performed in pigs and generated more than 100 families with dominantly or recessively inherited traits (66). In one family, a phenotype of hind limb paresis resembling PCS was observed. Within-family mapping of the variation underlying this phenotype revealed a 1 Mb region on SSC4 spanning 18 genes, among them the previously identified FBXO32. Subsequent analyses showed increased expression of FBXO32 mRNA and protein in three PCS-like piglets compared to three controls. Furthermore, MyoD and MyoG but not MYF5 were significantly downregulated in PCS-like piglets (Table 1). At histological level, interstitial fibrosis was observed in hind leg and longissimus muscles but not in fore limb muscles of affected piglets.

## Current Status

Decades of phenotypical description at different levels lead to a rather heterogeneous picture of the syndrome. Most work was concentrated on skeletal muscle tissue and myofibrillar hypoplasia of the affected hind legs, and also on the M. longissimus. MFH was therefore considered a common feature of splay leg in piglets. Besides MFH being a consequence of an immaturity of the muscular system at birth, an involvement of the nervous system was discussed as well. Specifically, a disturbed stimulus conduction, probably resulting from insufficient activity of acetylcholine and/or dysregulation of synaptic calcium metabolism, was further considered. In addition, metabolic disturbances like an impaired glycogen metabolism at the early postnatal period were discussed at length. However, many of the studies involved only a low number of piglets at different early postnatal time points and often lacked a clear phenotypic description.

A number of environmental factors are considered to be involved in or related to the expression of the PCS phenotype [reviewed in (24)]. This conclusion was derived from several studies reporting effects of nutrition, induced farrowing, housing conditions and diseases on the incidence of PCS. Insufficient choline supply of the sow was considered as cause of PCS by some authors (6, 44). This could lead either to reduced availability of choline phospholipids as membrane components or to a disordered signal transduction in skeletal muscle of the piglets (44). Indeed, dietary supplementation of choline to pregnant sows first seemed to prevent the occurrence of splay leg animals (6). PCS symptoms apparently disappeared after injection of the acetylcholine esterase blocker neostigmine-methylsulfate (21). However, these results could not be confirmed in other studies (48, 49). Furthermore, neither choline plasma levels nor activities of various choline esterases were found to be reduced in PCS piglets (44).

Induced farrowing as a common practice in the industrial pig production was also discussed as a possible cause of splay leg syndrome from the 1970's until recently (54). It was shown that induced farrowing increased the relative rate of piglet morbidity (74), but further studies found only a weak correlation between reduction of gestation length by induced farrowing and the incidence of PCS (54, 75, 76).

**Figure 2:**
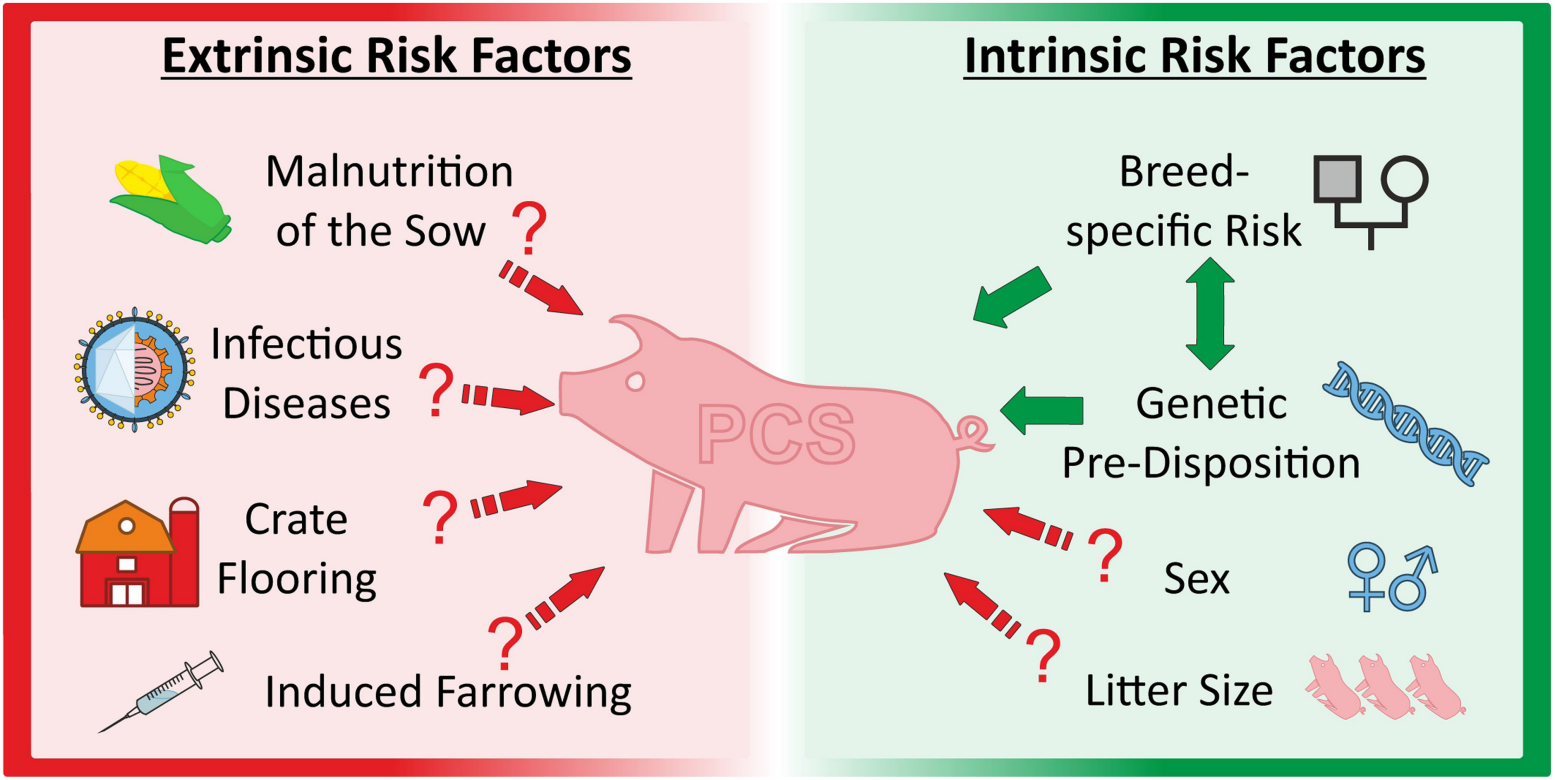
Overview about extrinsic and intrinsic factors discussed as causal and/or modulating factors for porcine congenital splay leg.

The porcine reproductive and respiratory syndrome (PRRS) was also suspected as being responsible for PCS. This virus infection results, among others, in reproductive failure including an increased number of abnormal piglets born. Vaccination against the PRRS virus was related to an immediate reduction of PCS piglets (77). However, there was no correlation found between anti-PRRSV vaccination and PCS incidence in a larger, more recent study (78). Although, vaccination significantly decreased reproductive problems, the number of PCS piglets did not differ between the treatments in three studied herds (78). In summary, environmental factors are likely to be involved in the modulation of PCS frequency and phenotypic expression but are unlikely as primary cause of the syndrome (Figure 2).

With the availability of molecular genetic methods in animal science, several studies aimed at identification of genetic causes for PCS. Besides global, transcriptional analyses of different muscles of affected piglets, also targeted, hypothesis-driven approaches toward putative functional candidates were conducted. As a result, several candidates were named but again, sampling of various muscles at differential time-points prevented broad comparability of the data. With the upcoming of GWAS as a powerful tool to identify phenotype-related genome variations, several chromosomal regions containing PCS-related SNPS were identified. The increasing detail of porcine genome annotation nowadays facilitates the listing of genes for further consideration.

Combining all published results, evidence for two putative pathological mechanisms evolved: Muscle fiber atrophy as suggested by the upregulation of the atrophy marker FBXO32 and delayed maturation of skeletal muscle at birth.

Latest research has presented two promising candidate genes potentially involved in either one of the proposed pathogenetic mechanisms. FBXO32 was not only identified in targeted as well as in global transcriptome analyses (5, 16) but its chromosomal region harbors genetic variants (66). HOMER1 was also found to contain specific SNPs using GWAS (17) which seem to alter the expression of certain isoforms *via* a modified intronic promotor (73). All studies were performed in different populations indicating a general validity (Tables 1, 2).

## Perspectives

A brief look into selected aspects of the proteins' physiology encoded by the most promising candidates underlines the opportunities opening up for future research.

First, FBXO32, also known as MAFbx, which is encoding an E3 ubiquitin ligase and therefore is considered as an early marker of atrophy in different species (5, 79, 80) might be causative for the observed retarded muscle maturation. Not only is the ubiquitin-proteasome pathway responsible for protein degradation which is typical for skeletal muscle atrophy (81), other results also suggest a negative regulation of MyoD1 and MyoG—both being master regulators of myogenesis—by FBXO32 (66). This underlines the importance of this factor for a multitude of processes in muscle development and growth. Although, no investigations on variations in the FBXO32 locus have been reported yet, future studies might still identify a genetic predisposition resulting in altered FBXO32 expression with implications for PCS. Furthermore, targeting upstream factors of FBXO32 like members of the FOXO family or the PI3K-Akt-pathway (16) might be useful for understanding the FBXO32-dependent aspect of PCS etiology.

Second, HOMER 1 as a member of the Homer protein family which is predominantly found in neural tissues is also expressed in muscle cells (82, 83) where it is directly involved in skeletal muscle differentiation. Homer proteins interact with numerous ligands [e.g., type 1 inositol trisphosphate (IP3) receptor, ryanodine receptor, and α-1D adrenergic receptor (84)]. With regard to muscle tissue, especially their binding capacity of intracellular ligand-gated Ca^2+^ channels like IP_3_R and RyR is a crucial aspect of Homer activity since activation of transcription factors such as MEF2 (85) or NFAT (86) are critical in muscle differentiation. Disturbances in the equilibrium of HOMER expression is therefore likely to impair such processes. Proper orchestration of muscular transcriptomes is essential for functional muscles. Its impairment might consequently result in the observed atrophy-like situation, and retarded muscle development. Because Homer proteins are critically involved in glutamatergic signaling, which is crucial for neuronal cells triggering and controlling myelination processes in oligodendroglia precursors (87, 88), it can be surmised that HOMER dysfunctions might also impair proper motor axon myelination (89). This may lead to improper muscle innervation as reported by Szalay et al. (41) for PCS piglets. Since certain SNPs have already been reported near or within the HOMER locus (17) and also seem to effectively modulate Homer isoform expression (73), this candidate might be the most promising in the search for the cause of the hereditary trait of PCS.

It should be noted that PCS is most likely a quantitative trait with at least a moderate number of genes involved. As summarized above, different physiological processes may be dysregulated in splay leg piglets. It is likely that further candidates will come into focus and add important facets to the picture. P311 (NREP) might be such an example since it is already known to be downregulated in splay leg piglets. This trans-differentiation factor is important for cell development and might therefore also fit the picture as a causing factor of MFH. Moreover, binding of HOMER to calcium channels regulates the activity of downstream transcription factors like NFAT which is critical for muscle differentiation (86). Interestingly, about one third of the differentially expressed genes identified in the study of Maak et al. (15) are direct targets of NFAT. Consequently, several pathways could be followed in parallel to contribute to a comprehensive elucidation of PCS' etiology and pathogenesis.

Future tasks to meet this aim could include the identification of causal variation leading to differential expression of certain candidates (e.g., FBXO32, NREP) as well as the functional characterization of identified SNPs (e.g., HOMER1). All candidates should be investigated in different populations to find general mechanisms causing PCS. Most importantly, clinical and histological parameters as well as experimental conditions have to be clearly defined and reported with sufficient detail to describe the phenotype as accurately as possible. Standardization of muscle sampling—anatomical location and timing—is of crucial importance for gene or protein expression studies. This would greatly enhance the comparability of different studies.

Since clinical and histological findings might still be highly biased by a plethora of vital parameters, isolation of muscle stem and progenitor cells, also known as satellite cells, would be a very useful tool in order to test the intrinsic differentiation capacity of cells derived from PCS piglets and controls *in vitro*.

Despite many uncertainties regarding the phenotypic and genetic background of PCS, still existing, recent studies have paved the road toward understanding this syndrome. Molecular genetics will enable pig breeders to select against porcine congenital splay leg in a foreseeable time.

## Author Contributions

SM and TS conceived the review. TS wrote the draft. TS, SM, and MR edited the manuscript. All authors contributed to the article and approved the submitted version.

## Conflict of Interest

The authors declare that the research was conducted in the absence of any commercial or financial relationships that could be construed as a potential conflict of interest.
